# True MEN1 or phenocopy? Evidence for geno-phenotypic correlations in MEN1 syndrome

**DOI:** 10.1007/s12020-019-01932-x

**Published:** 2019-05-01

**Authors:** Annamária Kövesdi, Miklós Tóth, Henriett Butz, Nikolette Szücs, Beatrix Sármán, Péter Pusztai, Judit Tőke, Péter Reismann, Mónika Fáklya, Géza Tóth, Anikó Somogyi, Katalin Borka, Annamária Erdei, Endre V. Nagy, Veronika Deák, Zsuzsanna Valkusz, Péter Igaz, Attila Patócs, Vince Kornél Grolmusz

**Affiliations:** 1 2nd Department of Medicine, Semmelweis University, Budapest, Hungary; 2 “Lendület” Hereditary Endocrine Tumors Research Group, Hungarian Academy of Sciences – Semmelweis University, Budapest, Hungary; 3 Department of Laboratory Medicine, Semmelweis University, Budapest, Hungary; 4 Kenézy Gyula Hospital, Debrecen, Hungary; 5 Markhot Ferenc Hospital, Eger, Hungary; 6 2nd Department of Pathology, Semmelweis University, Budapest, Hungary; 7 Division of Endocrinology, Department of Medicine, Faculty of Medicine, University of Debrecen, Debrecen, Hungary; 8 Kaposi Mór County Hospital, Kaposvár, Hungary; 9 1st Department of Medicine, University of Szeged, Szeged, Hungary; 10 MTA-SE Molecular Medicine Research Group, Hungarian Academy of Sciences – Semmelweis University, Budapest, Hungary

**Keywords:** Multiple endocrine neoplasia type 1, Neuroendocrine tumors, Gastroenteropancreatic neuroendocrine tumor, Phenocopy, Genotype–phenotype associations

## Abstract

**Purpose:**

Multiple endocrine neoplasia type 1 is a rare tumor syndrome caused by germline mutations of *MEN1* gene. Phenotype varies widely, and no definitive correlation with the genotype has been observed. Mutation-negative patients with MEN1-associated tumors represent phenocopies. By comparing mutation-positive and mutation-negative patients, we aimed to identify phenotype features predictive for a positive genetic test and to evaluate the role of *MEN1* mutations in phenotype modulation.

**Methods:**

Mutation screeening of *MEN1* gene by Sanger sequencing and assessment of clinical data of 189 consecutively enrolled probands and relatives were performed at our national and European Reference Center. Multiple ligation probe amplification analysis of *MEN1* gene and Sanger sequencing of *CDKN1B* were carried out in clinically suspicious but *MEN1*-negative cases.

**Results:**

Twenty-seven probands and twenty family members carried *MEN1* mutations. Five mutations have not been described earlier. Pronouncedly high number of phenocopies (>70%) was observed. Clinical suspicion of MEN1 syndrome emerged at significantly earlier age in *MEN1*-positive compared to *MEN1*-negative probands. Gastroenteropancreatic neuroendocrine tumors developed significantly earlier and more frequently in carriers compared to non-carriers. Probands with high-impact (frameshift, nonsense, large deletions) mutations, predicted to affect menin function significantly, developed GEP-NETs more frequently compared to low-impact (inframe and missense) mutation carriers.

**Conclusions:**

MEN1 phenocopy is common and represents a significant confounder for the genetic testing. GEP-NET under 30 years best predicted a *MEN1* mutation. The present study thus confirmed a previous proposal and suggested that GEP-NET under 30 years should be considered as a part of the indication criteria for *MEN1* mutational analysis.

## Introduction

Multiple endocrine neoplasia type 1 (MEN1) is a rare, autosomal dominantly inherited tumor syndrome caused by germline mutations of the tumor suppressor *MEN1* gene, with an estimated prevalence of 1–10/100,000 individuals [1, 2]. Most common manifestations include primary hyperparathyroidism (PHPT), pituitary adenomas (PA), and gastroenteropancreatic neuroendocrine tumors (GEP-NET). The tumors of the affected endocrine organs in MEN1 syndrome appear earlier than the sporadic ones. Their penetrance increases with age, although considerable phenotypic variability has been reported [3, 4].

Of the three major manifestations, PHPT has the highest penetrance and is considered to appear first in MEN1, although it often remains unrecognised [5]. Recent publications show, that functionally active GEP-NETs, initially frequently diagnosed as sporadic ones, lead to diagnosis of MEN1 in a remarkable proportion of patients [6]. Compared to sporadic tumors, MEN1-associated GEP-NETs are diagnosed 10 years earlier and often in a multiple form [5, 7], and their penetrance is as high as 80–90%, reaching nearly that of the parathyroid adenomas [6]. Non-functioning GEP-NETs are increasingly recognised due to advanced imaging modalities such as endoscopic ultrasound and thus became the most common type in MEN1 patients [8]. Although MEN1-associated GEP-NETs seem to have a low proliferation rate and long survival has been reported, they should be of particular attention, since they are still the principal cause of death in MEN1 patients [9, 10]. There are only a few studies comparing MEN1-associated versus sporadic GEP-NETs, and there are no unequivocal pieces of information about the possible differences regarding their prognosis [7, 9].

The criteria of diagnosis and the indication for *MEN1* mutation analysis have been described in the Endocrine Society guideline published in 2012 [8]. In 5–10% of MEN1 patients no mutation of the *MEN1* gene can be found. In these cases simultaneous development of endocrine tumors usually associated with *MEN1* mutations results in phenocopy [8]. Mutations of other genes might be responsible for a MEN1-like phenotype. Rare mutations of the *CDKN1B* gene encoding the cyclin dependent kinase inhibitor p27 causes the MEN1-like MEN4 syndrome [11]. Involvement of the *CaSR, AIP*, and *CDC73* genes was also demonstrated as a cause of MEN1-like syndromes [12].

Here, we present our experience with genetic diagnosis of MEN1 syndrome as a Hungarian national reference center from the last 17 years. *MEN1* mutation analysis was performed in all patients with clinical suspicion of MEN1 syndrome. *MEN1* mutation-positive and mutation-negative subjects were compared in order to identify predictive factors for true MEN1 cases.

## Subjects and methods

### Subjects

*MEN1* genetic test is available at our national referral center since 2001. A total of 189 patients, 134 unrelated probands and 55 family members of the mutation-positive pedigrees were examined for germline mutations. Between January 2001 and December 2017, patients were consecutively enrolled from all over Hungary and all data available were collected retrospectively. Of the 134 probands, 104 cases fulfilled the criteria of *MEN1* mutational analysis of the Endocrine Society published in 2012 [8]. All available first-degree relatives of the index cases genetically diagnosed with MEN1 were enrolled. Because of the limited availability of data regarding family history, the familial or sporadic origin of the disease could not be reliably determined in all cases. Clinical information was obtained from the responsible endocrinologists. Diagnosis of the manifestations was established according to the corresponding guidelines [8]. Further regular screening for tumors of the affected organs was performed in mutation-positive cases and in mutation-negative patients presenting with clinical MEN1 syndrome, in line with the widely accepted recommendations [3, 8]. Clinical data were studied together with laboratory, imaging, and histological results.

### Genetic analysis

Detection of disease-causing germline mutations of the *MEN1* gene was carried out using genomic DNA isolated from peripheral blood samples in all patients. The coding regions of the *MEN1* gene were PCR-amplified and were subjected to Sanger sequencing as described earlier [13, 14]. The new mutations found in *MEN1* gene were considered pathogenic based on their association with clinical MEN1 syndrome. Patients carrying a frameshift, nonsense, splice site mutation or large deletion were considered having a high-impact mutation. Those with a missense or inframe mutations were grouped as low-impact mutation carriers.

Due to the infrequent occurence of both large deletions in the *MEN1* gene and mutations of the *CDKN1B* gene, further genetic analysis was performed in those 15 suspicious, *MEN1* mutation-negative cases, who either developed all the three major manifestations, or presented two major manifestations before the age of 40 years. In these probands, MLPA analysis was performed to detect large deletions of the *MEN1* gene, using SALSA MLPA probemix kit P017-D1 according to the manufacturer’s instructions (MRC-Holland, Amsterdam, The Netherlands). For *CDKN1B* mutation analysis, the two coding exons of *CDKN1B* were PCR-amplified and directly Sanger sequenced using predesigned primer pairs (exon 1 forward primer (E1F): 5′ CGC TTT GTT TTG TTC GGT TT 3′; exon 1 reverse primer (E1R): 5′ ATA CGC CGA AAA GCA AGC TA 3′; exon 2 forward primer (E2F): 5′ TAA AAG CCA CTG GGG ATG AC 3′; exon 2 reverse primer (E2R): 5′ CAG TGC GTG CTC CTT TAG TG 3′). The PCR and sequecing protocols were described earlier [13–15].

### Statistical analysis

Dell Statistica (data analysis software system), version 13. (Dell Inc. (2016), software.dell.com.) was used for statistical analysis. Correlations between mutational status and clinical manifestations were calculated with *χ*^2^ and Fisher’s exact test. For examining the differences in age, Student’s independent samples’ *T*-test was used. For the examination of difference between age-related penetrances Kaplan–Meier curves were plotted and analyzed using log-rank (Mantel-Cox) test. In all comparisons, *p*-value < 0.05 was considered statistically significant.

## Results

### Results of genetic testing

A total of 189 patients, including 134 unrelated probands and 55 family members were enrolled in this study. *MEN1* mutation was identified in 47 cases: 27 probands and 20 family members. All of the 20 family members shared the same mutations as their index relatives. Of the 134 probands, the criteria for *MEN1* genetic testing were fulfilled in 104 cases (78 women and 26 men). No mutation was found in those patients who did not fit in the criteria of mutational analysis. Supplementary Table 1 contains detailed clinical and genetic data.

The 104 proband cases with clinical suspicion of MEN1 included 27 (26%) mutation-positive and 77 (74%) mutation-negative patients. We found 24 different *MEN1* gene mutations among these patients. Three patients carried c.1546_1547insC frameshift mutation in exon 10, two of them developed pancreatic NET under 30 years of age, the third patient had both metastatic pancreatic and bronchial NET. The c.202_206dupGCCCC mutation in exon 2 was identified in two probands. Ten mutations were described in our previous studies [13, 16], and further eight mutations have been reported formerly in the literature. To the best of our knowledge, of the 24 *MEN1* gene mutations, five (c.19 C>T, p.Gln7STOP in exon 2; c.1160delA in exon 8; c.1399delG in exon 10; c.166_167insA in exon 2 and c.168delC in exon 2) have not yet been published, thus these were regarded as novel mutations (chromatograms of the mutated sequences can be found in Supplementary Fig. 1). We found 15 mutation-negative cases (19.2%) that either presented all the three major tumors, or developed two major manifestations before 40 years of age. *CDKN1B* gene sequencing and MLPA analysis of the *MEN1* gene were carried out on these samples and in one case the deletion of exon 6 of *MEN1* was detected by MLPA.

Twelve mutation-positive index cases had familial MEN1 syndrome, while 11 had sporadic disease. In four cases, the familial or sporadic origin could not be determined. The family history in all *MEN1*-negative cases was negative, thus they are considered clinically sporadic. Thirty seven of the 77 mutation-negative probands (48.1%) fulfilled the criteria of clinical MEN1 syndrome, moreover, three of them developed all the three main manifestations of the syndrome (their age at the onset of PHPT was 48, 50 and 53 years; of PA 49, 50 and 53 years; of GEP-NET 46, 47 and 53 years, respectively). In accordance, the proportion of phenocopy within the sporadic patients fulfilling the criteria for clinical MEN1 syndrome (11 *MEN1*-positive sporadic patients and 37 *MEN1*-negative sporadic patients) was 77.1% (37/48).

### Indications of *MEN1* mutation analysis

**Fig. 1:**
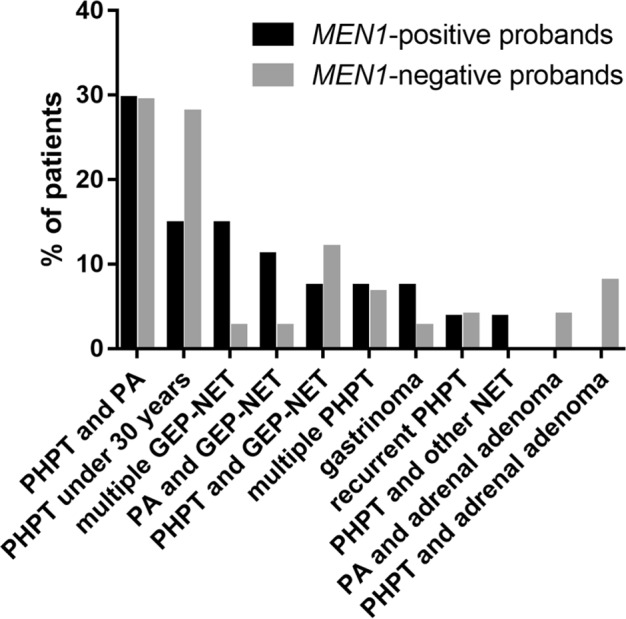
Indications for *MEN1* mutational analysis. The diagram shows manifestations that led to mutational analysis in mutation-positive (*n* = 27, marked with black) and mutation-negative (*n* = 77, marked with gray) probands. The criteria for analysis were defined according to the Endocrine Society guideline published in 2012 [8]. The group “multiple GEP-NET” includes both patients with multiple GEP-NETs of the same histological type and patients with multiple GEP-NETs of different subtypes. MEN1 multiple endocrine neoplasia type 1, PHPT primary hyperparathyroidism, PA pituitary adenoma, GEP-NET gastroenteropancreatic neuroendocrine tumor

The first clinical manifestation of the mutation-positive probands was PHPT and GEP-NET, both in 33.3% of patients, while PA presented in 26.0% as the first tumor. In one patient, bronchial NET appeared first. In *MEN1*-negative patients, PHPT, PA, and GEP-NET was the earliest tumor in 49.4, 27.3 and 13.0% of cases, respectively. Two mutation-negative patients developed adrenal adenomas before the appearance of PHPT, and in 7.8% information about the first tumor lacked. Figure 1 shows the indications for *MEN1* genetic testing. The most common indications were PHPT together with PA and PHPT under 30 years similarly in both *MEN1* mutation carriers and non-carriers. It is remarkable that multiple GEP-NETs presented quite often at the beginning of the clinical course of the disease, being a common indication for genetic testing in mutation carriers.

Of the 55 first-degree relatives, 20 carried *MEN1* mutations, and six of them did not have any signs or symptoms of MEN1. The youngest patient that underwent *MEN1* genetic testing was 6 years old, nonetheless, this patient inherited the mutation.

### Incidence and age-related penetrance of MEN1-associated tumors

The mean follow-up period lasted for 8.5 years. The mortality rate in *MEN1* mutation carriers and non-carriers was 14.8 (4/27) and 6.5% (5/77), respectively, which was not statistically significant (*p* = 0.186). However, three of the four MEN1 patients died because of GEP-NET (at the age of 35, 44 and 67 years; 10, 19 and 19 years after diagnosis, respectively), compared to only two of the five *MEN1*-negative probands (at the age of 44 and 56; 3 and 3 years after diagnosis, respectively). The suspicion of MEN1 syndrome emerged at significantly earlier age in *MEN1* mutation carriers compared to non-carriers (31.4 ± 12.6 vs. 40.2 ± 17.3 years, *p* = 0.019, Table 1). The incidence of GEP-NET was significantly higher already at initial presentation in mutation-positive compared to mutation-negative probands (44.4% vs. 20.8%, *p* = 0.017, not shown). Significantly higher prevalence of recurrent PHPT (55.6% vs. 9.1%, *p* < 0.001), PA (66.7% vs. 39.0%, *p* = 0.013), GEP-NET (70.4% vs. 23.4%, *p* < 0.001) and multiple GEP-NET (29.6% vs. 3.9%, *p* < 0.001) was observed in mutation-positive compared to mutation-negative cases (Table 1). Not only a significantly higher occurence of GEP-NET was associated with the mutant allele, but these tumors also developed significantly earlier in carriers (31.0 ± 12.2 vs. 45.9 ± 16.1 years, *p* = 0.004). More than the half of GEP-NETs in mutation-positive probands evolved under 30 years, compared to only 16.7% of GEP-NETs in non-mutant cases (*p* < 0.001). The combination of at least two of the three major tumors, as expected, correlated significantly with the carrier state. Any two of the three major tumors developed under 30 years of age represented a high predictive value (positive predictive value; PPV = 72.2%) for *MEN1* mutation. Apart from the coexistence of the three main manifestations, GEP-NET under 30 years of age best predicted a positive *MEN1* genetic test (PPV = 78.6%).

**Table 1:** Comparison of clinical characteristics of *MEN1* mutation-positive and mutation-negative probands

	Mutation-positive probands (*n* = 27)	Mutation-negative probands (*n* = 77)	*p*-value	% of mutation-positive probands (PPV)
Age at MEN1 syndrome suspicion* (years)	**31.4** ± **12.6**	**40.2** ± **17.3**	**0.019**	NA
PHPT	27 (100.0%)	69 (89.6%)	0.082	27/96 (28.1%)
Recurrent PHPT*	**15 (55.6%)**	**7 (9.1%)**	**<0.001**	**15/22 (68.2%)**
Age at PHPT (years)	33.4 ± 13.7	40.3 ± 17.9	0.082	NA
PHPT under 30 years	13 (48.2%)	26 (33.8%)	0.184	13/39 (33.3%)
PA*	**18 (66.7%)**	**30 (39.0%)**	**0.013**	**18/48 (37.5%)**
Age at PA (years)	29.8 ± 14.2	39.5 ± 16.5	0.053	NA
GEP-NET*	**19 (70.4%)**	**18 (23.4%)**	**<0.001**	**19/37 (51.4%)**
Multiple GEP-NET*	**8 (29.6%)**	**3 (3.9%)**	**<0.001**	**8/11 (72.7%)**
Age at GEP-NET*	**31.0** ± **12.2**	**45.9** ± **16.1**	**0.004**	NA
GEP-NET under 30 years* (years)	**11 (40.7%)**	**3 (3.9%)**	**<0.001**	**11/14 (78.6%)**
2 major manifestations under 30 years*	**13 (48.1%)**	**5 (6.5%)**	**<0.001**	**13/18 (72.2%)**
**PHPT** **+** PA*	**18 (66.7%)**	**26 (33.8%)**	**0.003**	**18/44 (40.9%)**
**PHPT** **+** GEP-NET*	**19 (70.4%)**	**12 (15.6%)**	**<0.001**	**19/31 (61.3%)**
**PA** **+** GEP-NET*	**12 (44.4%)**	**5 (6.5%)**	**<0.001**	**12/17 (70.6%)**
**PHPT** **+** **PA** **+** GEP-NET*	**12 (44.4%)**	**3 (3.9%)**	**<0.001**	**12/15 (80.0%)**

The table presents the frequency of the manifestations regarding genotype (i.e., mutation-positive or -negative), with the related *p*-values, in those probands who fulfilled the criteria of the mutation analysis (*n* = 104). The last column shows the positive predictive value (PPV) of each manifestation, that is: the proportion of mutation-positive probands among all patients carrying the manifestation. The manifestations signed with * mean significant associations. The group “multiple GEP-NET” includes both patients with multiple GEP-NETs of the same histological type as well as patients with multiple GEP-NETs of different subtypes

The bold values are significant

*MEN1* multiple endocrine neoplasia type 1, *PHPT* primary hyperparathyroidism, *PA* pituitary adenoma, *GEP-NET* gastroenteropancreatic neuroendocrine tumor, *PPV* positive predictive value, *NA* not applicable

**Fig. 2:**
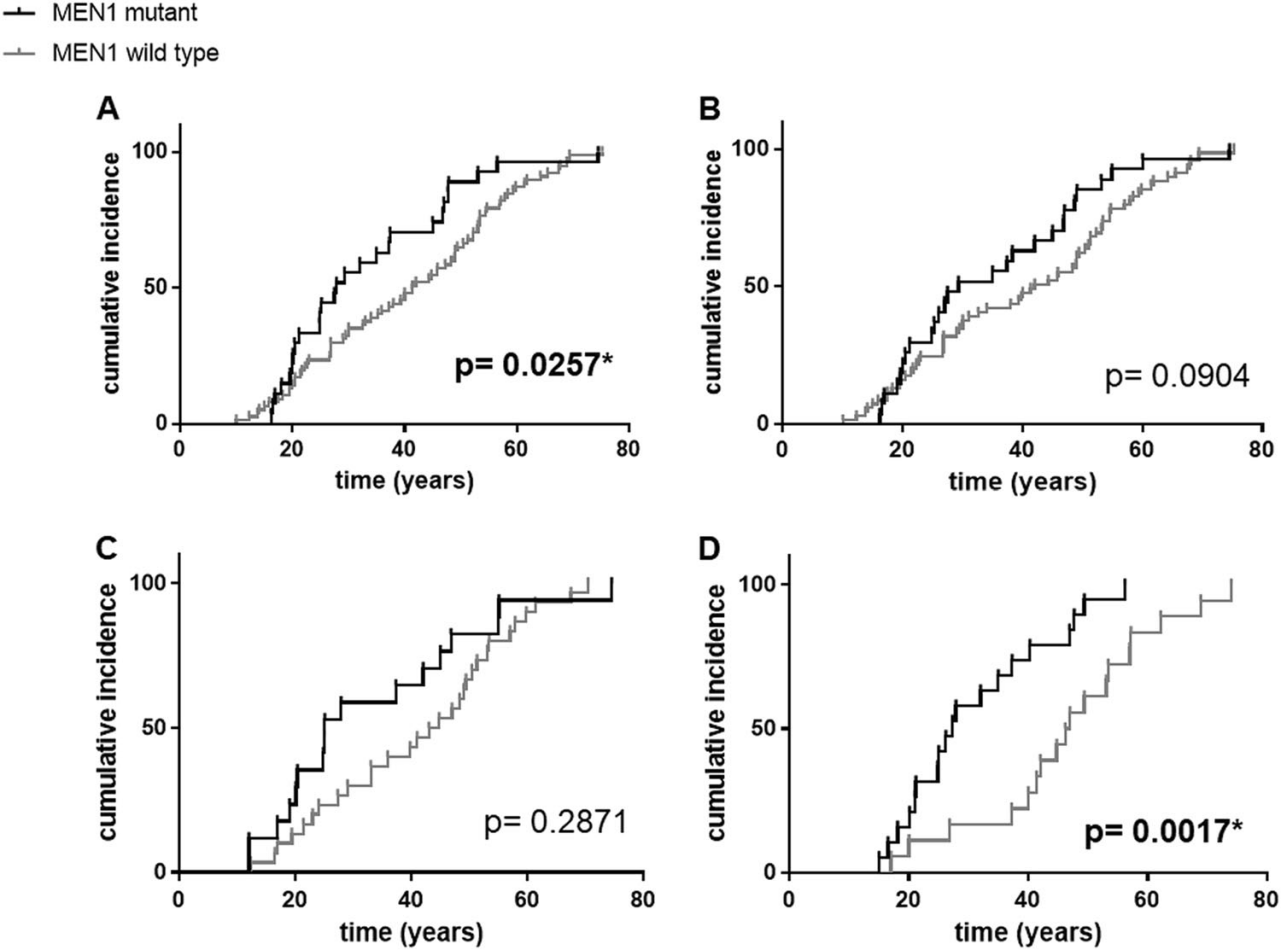
Age-related penetrance of the developement of MEN1 syndrome presumption (**a**) and the major manifestations: PHPT (**b**), PA (**c**), and GEP-NET (**d**) in *MEN1*-positive vs. *MEN1*-negative probands with the same manifestation. *P*-values marked with * mean significant associations. MEN1 multiple endocrine neoplasia type 1, PHPT primary hyperparathyroidism, PA pituitary adenoma, GEP-NET gastroenteropancreatic neuroendocrine tumor

The age-related penetrance of the development of MEN1 syndrome suspicion (according to the aforementioned criteria [8]) was significantly higher in mutation-positive compared to mutation-negative probands (Fig. 2); 50% penetrance was achieved at the age of 27.7 vs. 41.4 years, respectively. Similarly, significantly higher age-related penetrance of the development of GEP-NET was observed in mutation-positive patients, with 50% penetrance at the age of 26.8 compared to 46.2 years in mutation-negative probands.

### Histological type of tumors

The most common histological type of MEN1-associated GEP-NET was insulinoma (47.4%), followed by non-functioning pancreatic adenoma (31.6%) and gastrinoma (15.8%), with an overall penetrance of 33.3%, 22.2% and 11.1% among MEN1 patients, respectively. One patient developed glucagonoma and one patient was diagnosed with an ACTH-secreting NET. Three mutation-carrier probands had NETs of other origin: two patients had bronchial, and one had thymic NET. In contrast, insulinoma was present in only 22.2% of mutation-negative probands, while gastrinomas were more frequent (27.8%), whereas non-functioning tumors (22.2%) had similar frequencies. Three mutation-negative patients developed ileal carcinoids (16.7%), one patient had glucagonoma and one had VIPoma. None of the mutation-negative patients had either thymic or bronchial NET.

The majority of PAs in both mutation-positive and -negative probands was functional. The most common type of PA in MEN1-positive probands was prolactinoma (66.7%). Growth hormone (GH)—PRL-producing, GH-producing and follicle-stimulating hormone (FSH)—luteinizing hormone (LH)-producing adenomas were rare (11.1%, 5.6%, 5.6% of PAs, respectively). 11.1% of PAs were non-functioning. 33.3% of PAs of mutation-negative patients were GH-producing adenomas. Prolactinomas and non-functioning PAs were less frequent (both had 16.7% prevalence) and 6.7% of PAs produced adrenocotricotropic hormone (ACTH). In 26.7% of *MEN1*-negative cases, the data about hormonal activity was not available.

### Genotype–phenotype correlations

Following the concept of distinction between high- and low-impact mutations described above, we separated the mutation-positive probands into two groups to evaluate the possible influence of the mutation type on clinical outcome (Table 2). High-impact mutations included 12 frameshift mutations (44.4%, six deletions and six insertions), seven nonsense mutations (25.9%), one intronic splice-site mutation, and one large deletion. Low-impact mutations encompassed five missense mutations (18.5%) and one inframe deletion. GEP-NET had a significantly higher frequency among patients carrying high-impact mutations compared to those with low-impact mutations (81.0% vs. 33.3%, *p* = 0.044).

**Table 2:** Frequency of the manifestations in probands with high- and low-impact mutations, with the related *p*-values

	High-impact mutation carriers (*n* = 21)	Low-impact mutation carriers (*n* = 6)	*p*-value
Age at MEN1 syndrome suspicion (years)	30.6 ± 11.6	34.5 ± 17.6	0.543
PHPT	21 (100.0%)	6 (100.0%)	1.000
Recurrent PHPT	12 (57.1%)	3 (50.0%)	0.557
Age at PHPT (years)	33.1 ± 13.3	34.4 ± 16.9	0.856
PHPT under 30 years	11 (52.4%)	2 (33.3%)	0.362
PA	14 (66.7%)	4 (66.7%)	0.677
Age at PA (years)	27.9 ± 14.2	39.0 ± 12.0	0.230
GEP-NET*	**17 (81.0%)**	**2 (33.3%)**	**0.044**
Multiple GEP-NET	7 (33.3%)	1 (16.7%)	0.430
Age at GEP-NET (years)	31.5 ± 11.9	26.6 ± 12.5	0.602
GEP-NET under 30 years	10 (47.6%)	1 (16.7%)	0.189
2 major manifestations under 30 years	12 (57.1%)	1 (16.7%)	0.080
PHPT + PA	14 (66.7%)	4 (66.7%)	0.677
**PHPT** **+** GEP-NET*	**17 (81.0%)**	**2 (33.3%)**	**0.044**
PA + GEP-NET	10 (47.6%)	2 (33.3%)	0.443
PHPT + PA + GEP-NET	10 (47.6%)	2 (33.3%)	0.443

The manifestation signed with * mean significant associations. The group “multiple GEP-NET” includes both patients with multiple GEP-NETs of the same histological type and patients with multiple GEP-NETs of different subtypes

The bold values are significant

*MEN1* multiple endocrine neoplasia type 1, *PHPT* primary hyperparathyroidism, *PA* pituitary adenoma, *GEP-NET* gastroenteropancreatic neuroendocrine tumor

The most frequent location of mutations in the *MEN1* gene was exon 2 (40.7%), followed by exon 10 (18.5%). No mutations were detected in exons 5 and 7. No significant association was found between the affected exons and the clinical features.

## Discussion

Our study aimed to collect and analyze the clinical and genetic data of all Hungarian patients who underwent *MEN1* genetic testing at our national referral center. The estimated frequency of germline *MEN1* mutations in the Hungarian population according to our present data is 0.48/100,000 individuals, which is somewhat lower than expected [1]. However, compared to the recently published multicenter study of the Italian MEN1 database comprising 410 patients [17], the prevalence of *MEN1* mutations and the relative number of affected pedigrees in the whole population are similar to our findings. The mutation detection rate (the percentage of the mutation-positive cases among the index patients) in our study was 26%, similarly to the Swedish MEN1 cohort [18]. Additionally, of the 24 different *MEN1* mutations, five have not been documented yet.

Apart from the 27 genetically confirmed MEN1 index cases, 77 unrelated index patients with signs and symptoms resembling MEN1 syndrome were referred to our laboratory. Neither of these cases had MEN1 mutation. Hence, the percentage of sporadic MEN1 patients with phenocopy was 77.1% (37/48 cases), higher than previously estimated. Information about the proportion of MEN1 syndrome phenocopy is scarce, especially when distinguishing familial cases from sporadic ones. In these latter cases *MEN1* mutations occur far less frequently, in 33–65% of cases as previously reported [19]. As lately stated, such sporadic co-occurence of the main MEN1-associated endocrine tumors is presumably much more common than thought [20].

The most common indication for *MEN1* testing is the combination of PHPT and PA. They also occur frequently in the general population, thus their coexistence cannot be negligible. However, to the best of our knowledge, the exact frequency of the combination of these sporadic tumors is not known. Based on previous studies [20, 21], the co-occurence may be roughly estimated as high as 0.8–5.3/10,000 individuals, however, still considered a rare disease according to the Orphanet criterion [22].

Considering the low frequency of *MEN1* mutations and the high proportion of phenocopy in this population, one specific aim of the present study was to determine key factors which predispose to a positive result of the mutation screening by analyzing the differences between mutation-positive and mutation-negative patients. These findings may be useful for the clinician to identify those patients that most likely carry a *MEN1* mutation.

Since mutation-positive probands met the criteria for *MEN1* mutation screening at significantly earlier age than mutation-negative probands, we confirmed the previously described findings that younger age is a predisposing factor for a positive result during *MEN1* mutation analysis [2]. Moreover, the presence of any two of the three major tumors in patients under 30 years was highly predictive for a *MEN1* mutation. A considerable proportion of both *MEN1*-positive and *MEN1*-negative patients presented with PHPT either under 30 years or together with PA. This finding reflects the high prevalence and coincidence of these sporadic tumors in the general population, leading to phenocopy [8]; and also raises the issue of whether PHPT under 30 years implies a clear indication for *MEN1* mutational analysis.

The presence of GEP-NET implied the strongest predisposing factor regarding *MEN1* mutational positivity, already at initial presentation. Moreover, MEN1-associated GEP-NETs developed more than 15 years earlier than sporadic ones. Nonetheless, GEP-NET was the first clinical manifestation in one third of MEN1 probands. Consequently, of all the manifestations, the presence of GEP-NET under 30 years best predicted an underlying germline *MEN1* mutation. In accordance, Jensen et al. [23] found that MEN1-associated pancreaticoduodenal endocrine tumors (PDETs) are usually diagnosed one decade earlier than their sporadic counterparts. Furthermore, PDETs have been reported to be frequently the manifestation that leads to MEN1 diagnosis [5]. In line with previous studies, ENETS consensus guidelines already recommended *MEN1* genetic testing in patients with insulinoma before 20 years [24]. De Laat et al. [20] suggested lately, that mutational analysis should be extended to all patients with pancreatic NETs before 20 years of age. The present study, although with a limited number of cases, first evidenced this proposal: GEP-NET before 30 years should be especially considered to be included in the criteria of *MEN1* mutational analysis. This finding should certainly be confirmed on larger cohorts.

Five hundred and seventy-six different germline mutations of the *MEN1* gene were annotated between 1997 and 2015 [25, 26]. Concolino et al. [26] found a portion of 66.5% of frameshift, nonsense or splice site mutations, while Pardi et al. [27] and Lemos et al. [25] reported almost the same proportion as in our cohort (73% vs. 74%). There have been several attempts to find genotype–phenotype correlations regarding MEN1 syndrome. The functional effects of *MEN1* mutations have been widely investigated. One theory states that truncating (frameshift, nonsense) mutations may result in a consequent loss of functional domains, while the non-truncating (missense) forms cause inactivation of functionally critical amino acid residues [25]. Consequently, some studies report about the correlation between frameshift mutations and pancreatic neuroendocrine tumors [1, 28]. Instead, Machens reported no correlation between the type of mutation and the clinical features [29]. Accordingly with the recommendations of the American College of Medical Genetics and Genomics (ACMG) [30] and upon our results, *MEN1* genetic variants might also be categorized as low- and high-impact variants, which confer some limited prognostic differences. In our study, 11 out of 12 MEN1 patients with frameshift mutation had GEP-NET. High-impact *MEN1* mutation carriers were more likely to develop GEP-NETs than patients with low-impact mutations, resulting in a geno-phenotype correlation, which is of importance regarding the genetic counseling and the prognosis of MEN1 syndrome.

Our study presents some limitations, mainly as a consequence of the limited sample size. The clinical information may be incomplete in some cases, and the variable follow-up period did not allow us to draw firm conclusions regarding long-term disease course. The lack of symptoms and the limited availability of sensitive radiological methods presumably contributed to the underestimated proportion of non-functioning GEP-NETs in our cohort. Because of the limited possibility to test patients for large *MEN1* deletions with MLPA and for *CDKN1B* mutations, some of these mutations could have been missed.

In conclusion, we performed a comprehensive genetic and clinical analysis of the Hungarian MEN1 cohort. As a national and European Reference Center we have built the Hungarian MEN1 database, which may be of great interest in further work coordinated by the European Reference Network on Rare Endocrine Conditions (Endo-ERN, https://endo-ern.eu/). Based on our results a multicentric, international study has been initialised within the Endo-ERN in order to clarify whether our findings could be confirmed in large cohorts and consequently may lead to the modification of the criteria for *MEN1* mutation testing. The beneficial effect of routine screening of presymptomatic individuals has been recently debated [31] and even the timing of genetic testing of presymptomatic indviduals is questioned. The psychological distress and lower health-related quality of life in *MEN1*-positive individuals also indicate that this topic is highly relevant in everyday clinical practice [32]. We revealed a considerable proportion of patients with a high suspicion of MEN1 syndrome but without pathogenic *MEN1* mutation, resulting in phenocopy. Thus we aimed to find clinical features that most likely predict an underlying *MEN1* mutation by comparing mutation-positive and -negative patients. GEP-NET appeared significantly earlier and more frequently in *MEN1*-positive probands, and its development under 30 years best predicted a positive genetic test. Hereby we confirmed a lately raised suggestion and thus recommend extending the *MEN1* mutational analysis to all patients presenting GEP-NETs before 30 years. Moreover, MEN1 patients with high-impact *MEN1* mutations were more likely to develop GEP-NETs revealing an interesting geno-phenotypic association in MEN1 syndrome with potential prognostic consequences regarding genetic counseling. Finally we must add, as it has been previously confirmed many times, that despite the large number of negative results, genetic analysis is inevitable in suspicious cases of the dominantly inherited, highly penetrant MEN1 syndrome [2, 4, 33].

## Supplementary Information

Supplementary Figure

Supplementary Table
