# Supplementary figures and images for: True MEN1 or phenocopy? Evidence for geno-phenotypic correlations in MEN1 syndrome
Source: Endocrine. 2019 May 1;65(2):451–9. doi: 10.1007/s12020-019-01932-x (PMC6656790; doi:10.1007/s12020-019-01932-x)

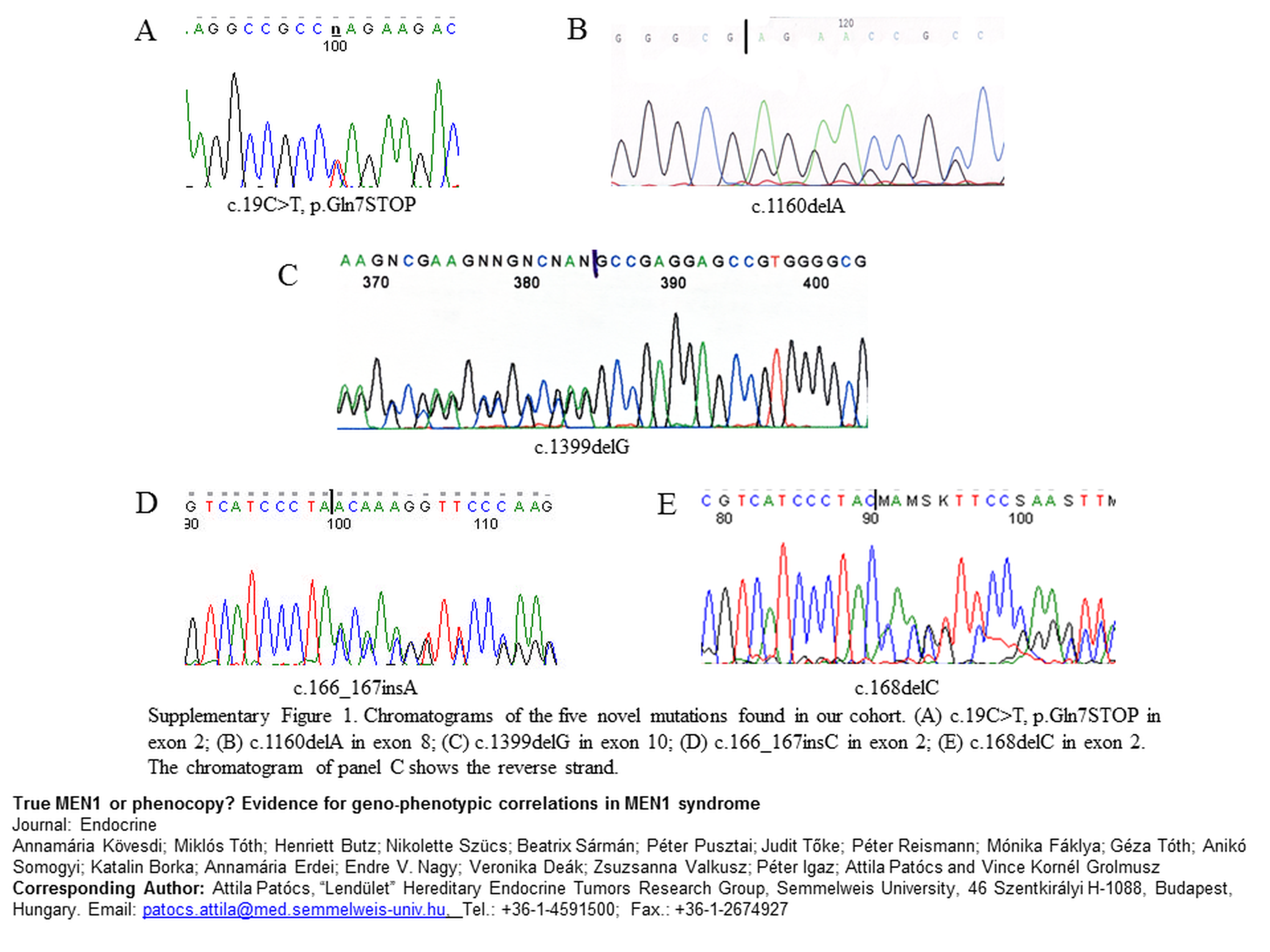

Supplement: Supplementary file 1 — Supplementary Figure [file 12020_2019_1932_MOESM1_ESM.tif]
